# Voltammetric Sensing of Chloride Based on a Redox-Active Complex: A Terpyridine-Co(II)-Dipyrromethene Functionalized Anion Receptor Deposited on a Gold Electrode

**DOI:** 10.3390/molecules29092102

**Published:** 2024-05-02

**Authors:** Kamila Malecka-Baturo, Mathias Daniels, Wim Dehaen, Hanna Radecka, Jerzy Radecki, Iwona Grabowska

**Affiliations:** 1 Institute of Animal Reproduction and Food Research, Polish Academy of Sciences, Tuwima Street 10, 10-748 Olsztyn, Poland; k.malecka@pan.olsztyn.pl (K.M.-B.); h.radecka@pan.olsztyn.pl (H.R.);; 2 Sustainable Chemistry for Metals and Molecules, Department of Chemistry, KU Leuven, Leuven Chem&Tech, Celestijnenlaan 200F, B-3001 Leuven, Belgiumwim.dehaen@kuleuven.be (W.D.)

**Keywords:** voltammetric sensor, anion recognition, host-guest interaction, terpyridine-Co(II)-dipyrromethene complex, chloride sensing

## Abstract

A redox-active complex containing Co(II) connected to a terpyridine (TPY) and dipyrromethene functionalized anion receptor (DPM-AR) was created on a gold electrode surface. This host-guest supramolecular system based on a redox-active layer was used for voltammetric detection of chloride anions in aqueous solutions. The sensing mechanism was based on the changes in the redox activity of the complex observed upon binding of the anion to the receptor. The electron transfer coefficient (*α*) and electron transfer rate constant (*k*_0_) for the modified gold electrodes were calculated based on Cyclic Voltammetry (CV) experiments results. On the other hand, the sensing abilities were examined using Square Wave Voltammetry (SWV). More importantly, the anion receptor was selective to chloride, resulting in the highest change in Co(II) current intensity and allowing to distinguish chloride, sulfate and bromide. The proposed system displayed the highest sensitivity to Cl^−^ with a limit of detection of 0.50 fM. The order of selectivity was: Cl^−^ > SO_4_^2−^ > Br^−^, which was confirmed by the binding constants (*K*) and reaction coupling efficiencies (*RCE*).

## 1. Introduction

The quantitative detection of anions is more challenging than that of cations due to their high hydration energy, complex geometry and pH-dependent properties [1,2]. Anionic species play a significant role in many chemical, biological, industrial, medical and environmental processes [3]. Therefore, the selection of appropriate detection techniques is in high demand [4]. Many analytical techniques, including absorption spectroscopy, fluorescence spectroscopy and electroanalytical methods, have been applied for sensing anions [5,6,7]. On the other hand, electrochemical sensors, including voltammetric, impedimetric, capacitive and potentiometric methods, thanks to their low price, sensitivity and scalability, have received increasing attention [7,8,9,10,11,12]. Most of these sensors contain synthetic receptors capable of reversibly binding anions based on non-covalent interactions, in particular hydrogen and halogen bonds [13]. These receptors could be immobilized on the surface of solid electrodes with recognition processes occurring at the solid/aqueous interface. In such a case, redox-active species could be either added to the sample solution [14] or functionalized with anion receptors and deposited on a solid substrate [15]. However, the development of electrochemical systems enabling the detection of anions in aqueous solutions, which consist of anion receptors linked in proximity with redox-active sites, is still of interest to the research community. The host-guest recognition phenomenon in such systems demands electrochemical communication between anion receptors and redox-active sites [16]. The binding mechanism between an anion receptor-bearing redox transducer and anions and subsequent signal transduction is still not fully understood [15].

Electrochemical sensors for the detection of chloride, thanks to their portability, relatively low cost, fast detection and user-friendliness, have the potential to be adjusted for on-site detection. Some voltammetric, potentiometric and colorimetric chloride sensors have been developed recently [17]. Just as an example, compact discs were used for preparation of working, counter and pseudo-reference electrodes and were set up in a three-electrode configuration cell, followed by using linear scan voltammetry (LSV) with a limit of detection (LOD) for chloride ions of 20 µM. In this case, excellent selectivity and reproducibility were reported [18]. Another chronoamperometric sensor for the detection of chloride ions was based on multiwalled carbon nanotubes (MWCNT), silver nanoparticles (AgNPs) and hydrophilic copper benzene 1,3,5-tricarboxylate (Cu-BTC) deposited on a flexible polytetrafluoroethylene (PTFE) substrate [19]. The electrochemical sensing of chloride has also been reported on glassy carbon electrodes (GCE) modified with AgNPs decorated into cavities of a zeolitic imidazolate framework 8 (ZIF-8). The reported sensor showed excellent stability, reproducibility and selectivity towards chloride ions with a LOD of 0.61 µM [20]. All of these examples concern the use of silver-based electrodes to detect Cl^−^. Another type of sensor is based on three screen-printed electrodes (SPE): a working, an auxiliary (both carbon paste electrodes, CPEs), and a pseudo-reference Ag/AgCl paste-based electrode. The carbon paste of the working SPE was filled with potassium ferricyanide, potassium ferrocyanide and ferrocenemethanol (FcMeOH). The systematic shift of the voltammetric peak potential of FcMeOH upon contact with chloride ions displayed the lowest LOD of 10 mM for Cl^−^ [21]. The LOD was further improved for this system using Gore-Tex^®^ support, up to 0.2 mM for chloride ions [22]. The systems in which redox-active species are co-immobilized together with anion receptors on the solid substrate and the detection of anions occurring in water are scantly present in the literature [23]. Several reports have been presented by the group of Beer and co-workers [7,15], but these systems were studied in non-aqueous solutions.

In our previous work, we proposed an electrochemical anion sensor made of electroactive dipyrromethene (DPM) complexes with Co(II) or Cu(II) incorporating anion receptor (AR), deposited onto a gold electrode ((Au/DPM/Co(II), Cu(II)/DPM-AR)) [23]. This system is based on a bifunctional receptor composed of receptor-binding sites and redox-active centers. The binding of anions to receptor molecules influences changes in electron transfer reactions, which is the basis of the generation of the analytical signal. Based on the previously obtained results, we can conclude that Co(II) redox centers showed better selectivity towards Cl^−^ than Cu(II) centers. In the present work, we have tested terpyridines as a possible coordination ligand [24]. The complexes of Co(II) with TPY and a dipyrromethene functionalized anion receptor (DPM-AR) were deposited on a gold electrode for sensing Cl^−^ using SWV. The chloride ions are very common in the environment due to intensive human industrial activity. High chloride content is dangerous to human life; therefore, it is important to carefully monitor the chloride content in water. There is a need to develop new methodologies that are characterized by high sensitivity, high throughput and high anti-interference ability [25]. Our system offers ultra-high sensitivity for chloride anions, with a detection limit of 0.5 fM. Therefore, the innovation of the system presented is related to the possibility of detecting these anions in very diluted samples, which can possibly reduce the matrix effect (possible interferences).

## 2. Results and Discussion

### 2.1. The Procedure of Au/MBL, AHT/TPY/Co(II)/DPM-AR Layer Preparation

**Scheme 1:**
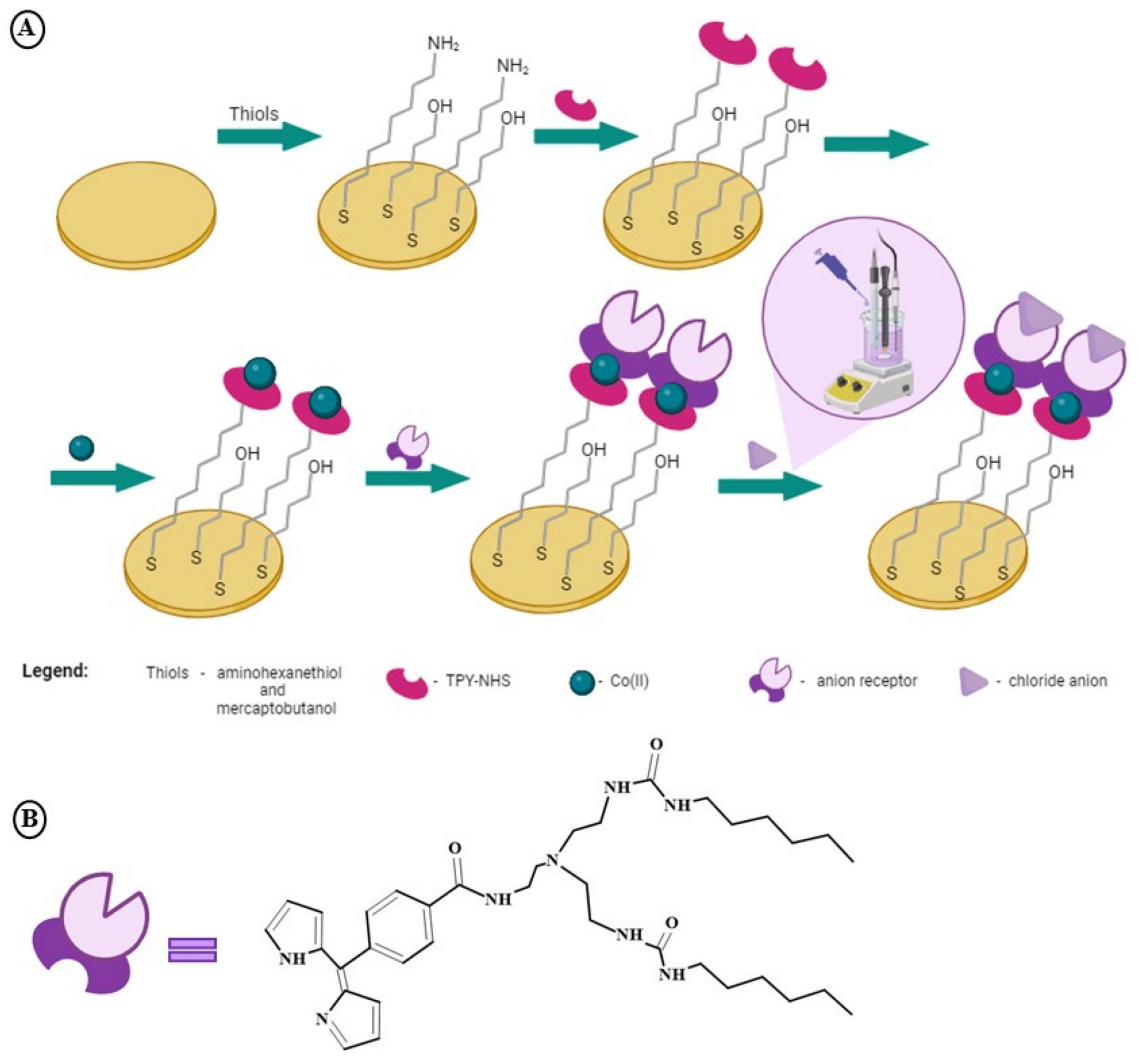
(**A**) Schematic representation of the gold electrode modification with the redox-active layer and anion receptor; (**B**) Structure of the anion receptor (DPM-AR).

The sensing layer was prepared based on a step-by-step gold electrode modification procedure. The first step was to simultaneously immobilize 6-amino-1-hexanethiol (AHT) and 4-mercapto-1-butanol (MBL) onto a gold electrode. In this way, the amino groups were available to create a covalent bond with TPY-bearing N-hydroxysuccinimide (NHS) groups. Upon complexation of TPY with Co(II) cations, DPM-AR molecules were attached to the electrode as the final step. An illustration of the redox-active sensing layer preparation (Au/MBL, AHT/TPY/Co(II)/DPM-AR) is presented in Scheme 1A. The chemical structure of the DPM-AR is shown in Scheme 1B. The system developed in this way contains a bifunctional receptor composed of a Co(II) redox-active center and an anion receptor. As a result of molecular recognition between the anion receptor and a specific anion, changes in the electrochemical activity of the redox-active Co(II) centers are registered by electrochemical methods, which are the base of the generation of the analytical signal. The bare gold electrodes were electrochemically characterized before modification. The electroactive surface area (*A_eas_*) and the roughness factor (*RF*) were determined, which were 0.32 ± 0.02 cm^2^ and 10.34 ± 0.52, respectively (Equations (S1)–(S3), Supplementary Materials) [26,27].

### 2.2. The Electrochemical Characterization of the Au/MBL, AHT/TPY/Co(II)/DPM-AR Layer

**Figure 1:**
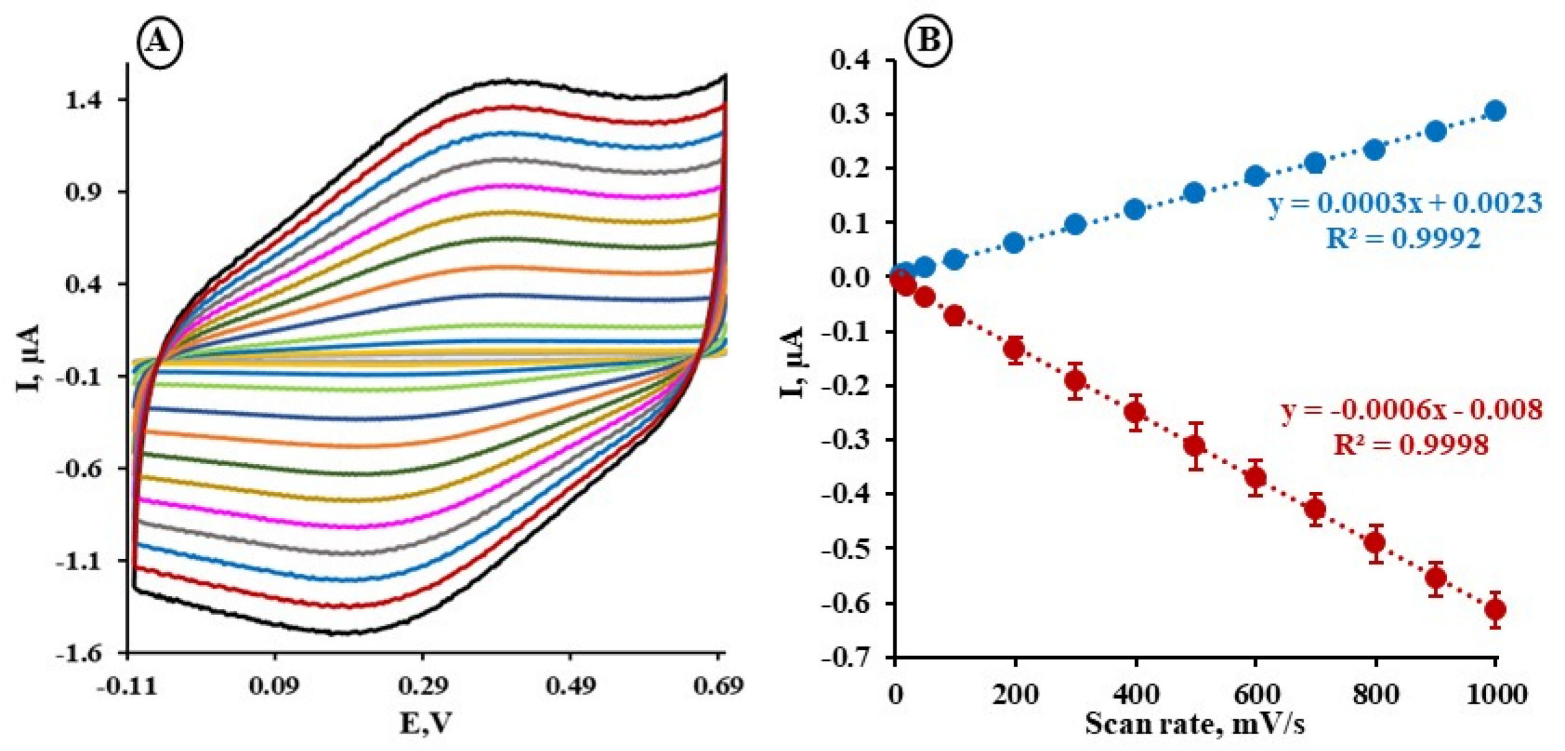
(**A**) An example of CV curves obtained for the gold electrode surface modified with MBL,AHT/TPY/Co(II)/DPM-AR layer, (**B**) plot of (●, I_pa_) anodic and (●, I_pc_) cathodic peak current vs. potential scan rate (0.050–1.0 V/s). Buffer composition: 0.1 M NaNO_3_ + 0.01 M H_3_BO_3_ at pH 4.0.

The Au/MBL, AHT/TPY/Co(II)/DPM-AR redox-active layer was initially electro-chemically characterized by CV experiments conducted at different scan rates from 0.05 to 1.0 V/s in a 0.1 M NaNO_3_ + 0.01 M H_3_BO_3_ buffer at pH 4.0 (Figure 1A). The composition and pH of the supporting electrolyte were thoroughly optimized in our previous studies [23]. At the scan rate of 0.1 V/s, the reduction and oxidation peaks were registered at the potentials of 309 ± 11 mV and 383 ± 16 mV, respectively. These results therefore demonstrate quasi-reversible electrode reactions for Co(II) deposited on the gold electrode surface. On the other hand, the linear relationships of reduction and oxidation peak currents vs. scan rate confirm the presence of Co(II) centers on the surface of the gold electrodes (Figure 1B). The cathodic peak is more pronounced, so the slope of the cathodic peak current was larger than the slope of the anodic peak current. 

The number of anion receptor molecules deposited on the gold electrode surface could be estimated based on the quantity of charge involved in the Co(III)/Co(II) reduction processes registered in CV. Surface coverage (Γ) of redox-active centers was calculated based on Equation (S4) (Supplementary Materials) [28] and is 2.4 ± 9.5 × 10^−11^ mol/cm^2^. As expected, this value is in a similar range as calculated for analogous systems in our previous work [29].

The values of the electron transfer coefficient (α) and the electron transfer rate constant (k_0_ [s^−1^]) for the Au/MBL, AHT/TPY/Co(II)/DPM-AR layer were calculated based on the generally applicable Laviron’s formula (Equations (S5) and (S6), Supplementary Materials) and amounted to 0.53 ± 0.01 and 0.99 ± 0.03, respectively. The value of α is close to 0.5, indicating an equal share of the total free energy of activation for electron transfer between the anodic and cathodic steps [30,31].

### 2.3. The Electrochemical Detection of Chloride Using the Au/MBL, AHT/TPY/Co(II)/DPM-AR layer

**Figure 2:**
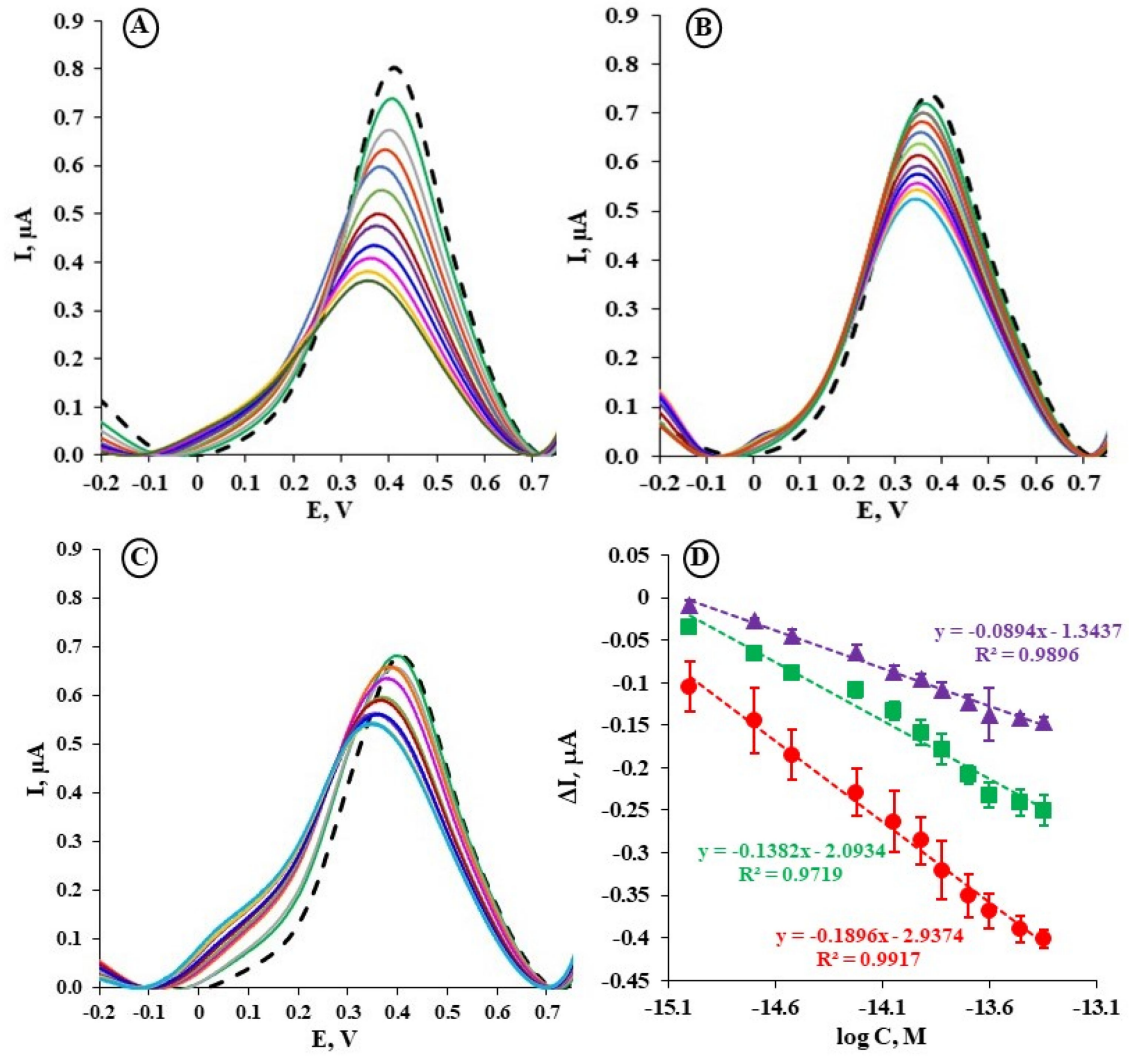
Representative SWVs recorded for the Au/MBL, AHT/TPY/Co(II)/DPM-AR layer in the presence of (**A**) chloride, (**B**) sulfate and (**C**) bromide in 0.1 M NaNO_3_ + 0.01 M H_3_BO_3_ at pH 4.0. (**D**) Redox current differences ΔI = I_n_ − I_0_, µA for the Au/MBL, AHT/TPY/Co(II)/DPM-AR in the presence of (●) chloride, (■) sulfate and (▲) bromide in 0.1 M NaNO_3_ + 0.01 M H_3_BO_3_ at pH 4.0 (*n* = 5 ÷ 6).

The electrochemical detection of chloride using the Au/MBL, AHT/TPY/Co(II)/DPM-AR layer was carried out based on SWV experiments. The gold electrode modified with the MBL, AHT/TPY/Co(II)/DPM-AR layer was characterized by the peak current and position of 0.78 µA and 410 mV, respectively, registered in 0.1 M NaNO_3_ + 0.01 M H_3_BO_3_ at pH 4.0 (Figure 2A, dashed black line). Initially, the concentration range of the system response towards Cl^−^ was optimized to acquire optimal analytical parameters for the presented system [23,24]. The procedure of optimizing the concentration range was started from conducting experiments of detection of the chloride anions by the system proposed in the pM concentration range. However, due to the large decrease in the current caused by the interaction of Cl^−^ with DPM-AR in this concentration range, in the next steps, the concentration range was lowered by one order of magnitude at a time until the system showed a linear change in the ΔI (µA) vs. log C (M). Finally, the concentration range between 1 and 45 fM was selected for the study of these interactions.

When the MBL, AHT/TPY/Co(II)/DPM-AR modified gold electrodes were in contact with growing concentrations of Cl^−^ (Figure 2A), a gradual decrease in the relative Co(II)/Co(III) peak current intensity was observed up to ΔI equal about −0.41 ± 0.01 µA (Figure 2D). The current decrease was assisted by a peak potential shift towards lower values. For the highest concentration of chloride (45 fM), the potential shift was −48.6 ± 2.3 mV (Figure S1, Supplementary Materials). A similar phenomenon, namely a cathodic shift ΔE [mV] of the redox-active anion receptor upon anion binding, was observed by other authors [15]. 

The selectivity of the sensor is an important factor influencing its analytical performance. To evaluate the selectivity of this layer, the two target anions, sulfate and bromide, were tested separately under the same conditions. Significantly smaller decreases in the Co(II)/Co(III) peak current, compared to Cl^−^, were observed for the Au/MBL, AHT/TPY/Co(II)/DPM-AR upon interaction with 45 fM of SO_4_^2–^ and Br^–^, up to −0.25 µA ± 0.02 and −0.14 ± 0.01 µA, respectively (Figure 2B,C). Additionally, the shift of the potential for sulfate and bromide was similar, about −23 mV (Figure S1, Supplementary Materials), which is approximately two times smaller than for chloride. An additional parameter determined the selectivity of the sensor—the response ratio R_i,j_ was calculated according to Equation (S7) (Supplementary Materials). The low values of R_i,j_ (below 1.0) of 0.73 and 0.47 (Supplementary Materials, Table S1) for interfering anions showed that the selectivity of chloride towards sulfate and bromide is very high [32]. The selectivity factor α_f_ of the proposed sensor was estimated by Equation (S8) (Supplementary Materials) to be equal to 2.1 (SO_4_^2−^) and 3.9 (Br^−^) [33]. 

The mechanism of the high selectivity for Cl^−^ anions is related to the higher binding constant of Cl^−^ with the anion receptor. The corresponding thiourea derivatives are known as effective receptors for chloride [34]. The complexation of the chloride to the anion receptor influences the redox reaction of the cobalt redox center. Different possible pathways of the reaction coupling realization include those through space electrostatic interaction between redox centers and complexed anions, bond communication, or conformational changes of the redox centers influenced by the complexation process. All these possible phenomena could cause changes in electron transfer efficiency to/from the electrode and reflect the reaction coupling efficiency (RCE) [16]. 

The linear relationships between the relative values of the current decrease of Co(II)/Co(III) peak current and concentration of chloride ions were observed over the entire range of analyzed chloride, from 1 to 45 fM (Figure 2D). On the other hand, the LOD was calculated using Equation (S9) and was equal to 0.50 fM (Supplementary Materials) [35]. 

**Scheme 2:**
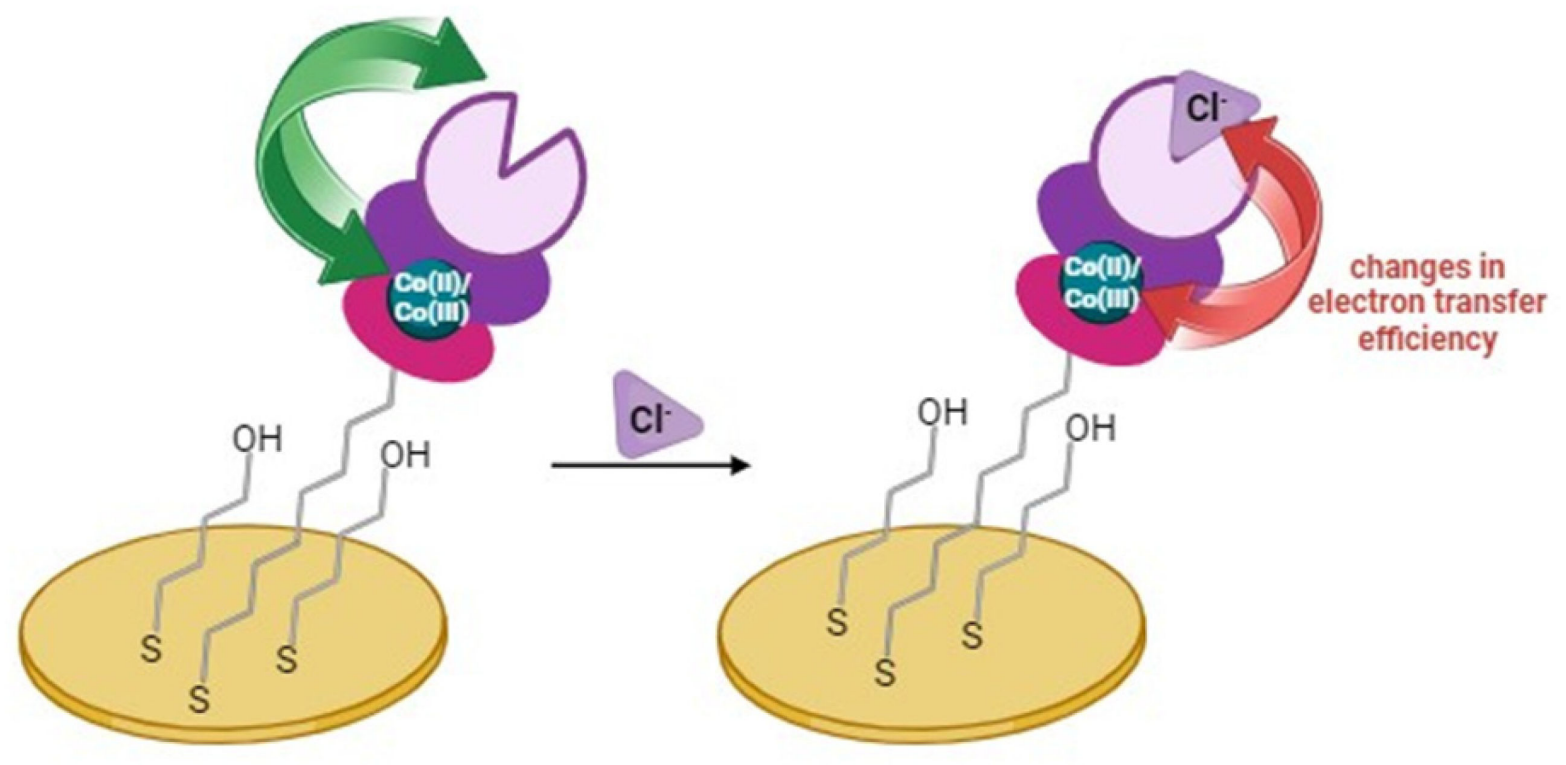
Schematic representation of the sensing principle.

Over the last decade, a few reports on the electrochemical detection of chloride anions in aqueous environments have been published in the scientific literature [18,19,20,21,22,23,36,37,38,39,40]. Some examples collected in Table 1 clearly show that the presented system has better sensitivity (fM range) than most of the literature references. The LODs of most sensors displayed in Table 1 are in the micromolar or lower range for chloride determinations. Only one example, which is based on a redox-active receptor deposited on the gold electrode surface, allowing for the determination of Cl^−^ in the pM range in aqueous solutions, has been presented till now [23]. Our results clearly show that changing the dipyrromethene to terpyridine to form a redox-active complex with Co(II) improved the LOD of Cl^−^ by almost three orders of magnitude. This system is based on a bifunctional receptor molecule that contains two elements in one, namely binding sites of AR and a TPY/Co(II)/DPM redox-active center. The electron transfer reactions of the redox centers are coupled with the complexation reactions of the receptor (Scheme 2). The specific mechanism is worth further exploration. However, there are some possible explanations that could be related to the differences in thermodynamically important stability constant of complexes TPY/Co(II)/DPM or DPM/Co(II)/DPM or the differences in the conformational induced perturbation of redox centers caused by the complexation of the anion to the anion receptor [16]. Moreover, it has been reported that terpyridines are π-acceptors and stabilize the metals in a lower oxidation state, which could be an additional motive for the difference in the obtained LOD in both cases. 

The association constant (*K_A_*) of the AR with chloride was calculated using a Langmuir isotherm approach [29] and was found to be 2.78 ± 0.21 × 10^13^ M^−1^. This approach is based on the linearization of the Langmuir isotherm, according to Equation (S10). The linear relationship of (*I_n_* − *I*_0_)/*I*_0_ vs. *C* [*M*] is shown in Figure S2, Supplementary Materials. Hence, the affinity interaction between AR and chloride ions was described by a dissociation constant *K_D_* of 6.13 ± 0.45 × 10^−14^ M (Equation (S11), Supplementary Materials). Then, the thermodynamic parameter Δ*G* (change in Gibb’s free energy) using Van ’t Hoff (Equation (S12), Supplementary Materials) [41] was calculated and was equal to −69.6 ± 2 kJ/mol, which means spontaneous interaction of anion receptor and chloride (Table S2, Supplementary Materials).

Interfacial binding constants (*K*) for the formation of complexes of the target anions with the AR were also estimated. The detailed procedure for these calculations is demonstrated in the Supplementary Materials (Equation (S13), Figure S3) [42]. The obtained results clearly show that the affinity for chloride (20 × 10^+13^ M^−1^) is the strongest, and the values of the sulfate (5.13 × 10^+13^ M^−1^) and bromide (2.13 × 10^+13^ M^−1^) binding constants were an order of magnitude lower (Table S3). The explanation for such high binding constants (*K*) is the fact that the anion recognition process occurs at the interface between the aqueous solution and the modified electrode surface [23].

The reaction coupling efficiency parameter (*RCE*) is the next factor, which was calculated to support and confirm the obtained results. It provides information about the effectiveness of anion binding to the receptor, calculated according to Equation (S14) (Supplementary Materials) [43]. CV curves recorded at anion concentrations of 0 and 15 fM were selected to calculate the *RCE* parameter. The obtained *RCE* values are 5.2 ± 0.5, 2.3 ± 0.6, and 1.3 ± 0.3 for chloride, sulfate and bromide, respectively (Table S3). The *RCE* value for Cl^−^ is higher than for SO_4_^2–^ and Br^-^, which indicates that chloride complexation is more efficient [23].

### 2.4. The Repeatability, Reproducibility and Stability of the Sensing Layer

**Figure 3:**
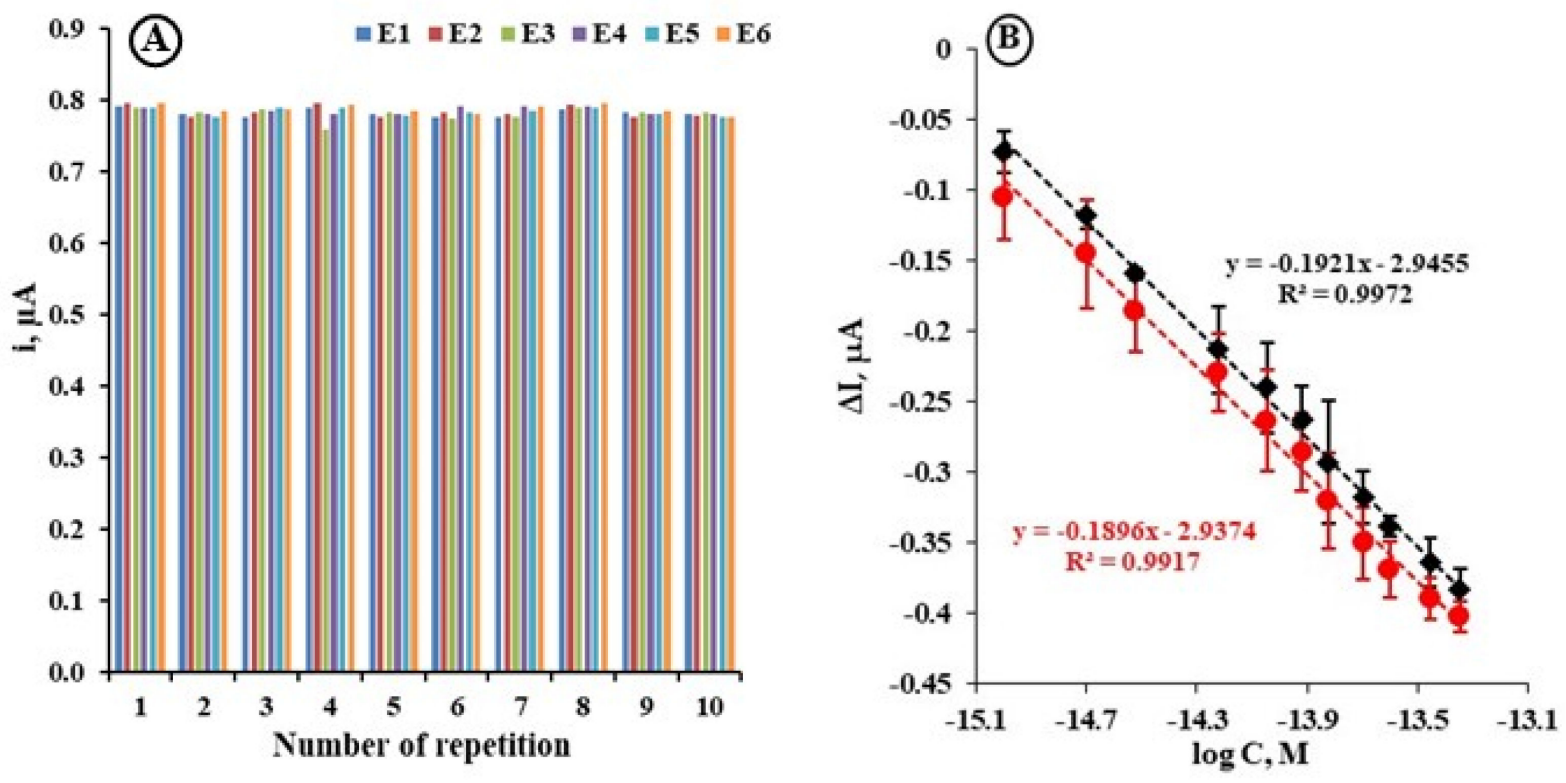
(**A**) Results of repeatability and reproducibility studies obtained for the gold electrode surface modified with the MBL, AHT/TPY/Co(II)/DPM-AR in 0.1 M NaNO_3_ + 0.01 M H_3_BO_3_ at pH 4.0. (**B**) Redox current differences ΔI = I_n_ − I_0_, µA recorded using SWV for Au/MBL,AHT/TPY-Co(II)/DPM-AR in the presence of (●) chloride measured directly after modification and (♦) chloride measured 7 days of storage at 4 °C in 0.1 M NaNO_3_ + 0.01 M H_3_BO_3_ at pH 4.0 (*n* = 3).

The sensing layer of Au/MBL, AHT/TPY/Co(II)/DPM-AR was also characterized by repeatability, reproducibility and stability over time. Repeatability and reproducibility were then assessed with relative standard deviations (RSD) of 2.9% and 4.7%, respectively (Figure 3A). The stability of the sensor was examined by recording SWV curves in the presence of Cl^−^ anions in the concentration range from 1 fM to 45 fM after 7 days of storing gold electrodes modified with TPY/Co(II)/DPM-AR at 4 °C. Stability studies show that after 7 days of storage, the sensor response decreases by ~9.5% (Figure 3B).

## 3. Materials and Methods

### 3.1. Materials

The DPM-AR (Scheme 1B) and the terpyridine-NHS (TPY–NHS) were synthesized at the Department of Chemistry, KU Leuven, Leuven. The procedure for synthesis was briefly described in the Supplementary Materials (Scheme S1) [23]. AHT, MBL, cobalt (II) acetate Co(OAc)_2_, dichloromethane (DCM), sodium chloride (NaCl), sodium sulfate (Na_2_SO_4_), sodium bromide (NaBr), sodium nitrate (NaNO_3_) and boric acid (H_3_BO_3_) were purchased from Sigma-Aldrich (Poznań, Poland). Potassium hydroxide (KOH), sulfuric acid (H_2_SO_4_), nitric acid (HNO_3_) and methanol (MeOH) were obtained from Avantor Performance Materials (Gliwice, Poland). All reagents were of analytical grade. All aqueous solutions were prepared with deionized and charcoal-treated water (resistivity of 18.2 MΩ cm) purified with a Milli-Q reagent grade water system (Millipore, Bedford, MA, USA). These solutions were deoxygenated by purging with nitrogen (ultrapure 6.0, Air Products, Warszawa, Poland) for 15 min. 

### 3.2. Electrochemical Measurements

All electrochemical measurements were performed with a potentiostat-galvanostat AutoLab (Eco Chemie, Utrecht, The Netherlands) with a three-electrode configuration system. Potentials were measured vs. a silver chloride (Ag/AgCl) electrode, and a platinum wire was used as an auxiliary electrode. The voltammetric experiments were carried out in an electrochemical cell with a 10 mL volume. CV measurements were performed in the potential window from −100 mV to 700 mV with different scan rates: 10, 20, 50, 100, 200, 300, 400, 500, 600, 700, 800, 900 and 1000 mV·s^−1^. SWV was performed with a potential from −100 mV to 700 mV with a step potential of 1 mV, a square wave frequency of 50 Hz, and an amplitude of 50 mV. 

All measurements were conducted in the presence of an aqueous solution of 0.1 M NaNO_3_ and 0.01 M H_3_BO_3_ (pH 4.0) as a supporting electrolyte. The influence of the pH on the response of this system in both the absence and presence of Cl^−^ anion was studied by varying the pH of the supporting electrolyte (0.1 M NaNO_3_ + 0.01 M H_3_BO_3_) in a 4.0–7.0 range. The results showed that well-defined quasi-reversible peaks for Co(II)/Co(III) were obtained at pH 4.0. The reversibility of the redox reaction and the peak current decrease with an increase in the pH of the solution. Furthermore, at pH 4.0 the tertiary nitrogen atom of the anion receptor is protonated causing a stronger electrostatic interaction between the anion receptor and the selected anions (Cl^−^, SO_4_^2−^, and Br^−^). Therefore, in order to get a stronger response and sensitivity of the sensing system, pH 4.0 was chosen as the optimum pH value for all the experiments [23]. Sensing experiments were carried out by adding target anions to the electrochemical cell. The measuring conditions for chloride recognition were experimentally selected in our previous work [23]. The analytical signal of the electrode was expressed as: ΔI = I_n_ − I_0_, where I_n_ is the peak current measured in the presence of the particular concentration of the analyte (anion) and I_0_ is the peak current before adding the analyte to the pure supporting electrolyte. 

### 3.3. Fabrication of Sensing Layer for Detection of Chloride

Gold disk electrodes with a diameter of 2 mm (Bioanalytical Systems (BASi), West Lafayette, IN, USA) were used for all experiments. The preparation of gold electrode surfaces was performed according to an already published protocol [44]. Briefly, these electrodes were initially manually polished with alumina slurries (0.3 μm and 0.05 μm, Alpha and Gamma Micropolish, Buehler; Lake Bluff, IL, USA). The polished electrodes were then electrochemically cleaned by CV in 0.5 M KOH and then in 0.5 M H_2_SO_4_. Before modification, the electrode surfaces were refreshed with 0.5 M KOH. After electrochemical pre-treatment, each electrode was rinsed with Milli-Q water and kept in water (for a few minutes until the next step) to avoid air contamination. Immediately after cleaning, the electrodes were repeatedly rinsed with Milli-Q water, MeOH, and a mixture of MeOH and DCM (volume ratio 1:1) and immersed in modification solutions. Preparation of the layer has been illustrated in Scheme 1A and is as follows:

1. The gold electrodes were immersed in a mixture of 0.01 mM AHT and 1 mM MBL—3 h at room temperature (RT), DCM:MeOH (1:1, *v*/*v*); after modification, electrodes were carefully rinsed with a mixture of DCM:MeOH;
2. The reaction between the amine groups of AHT and NHS of 0.1 mM TPY-NHS during 1 h, RT, DCM:MeOH (1:1); after this step, electrodes were carefully washed with a mixture of DCM:MeOH;
3. The complexation of Co(II) ions was achieved by immersing electrodes in 1 mM of Co(OAc)_2_—1 h, RT, DCM:MeOH (1:1); after modification, electrodes were carefully rinsed with a mixture of DCM:MeOH;
4. The coordination sphere of the Co(II) metal ions was closed by 0.1 mM DPM-AR—1 h, RT, DCM-MeOH (1:1); after modification, electrodes were carefully washed with: a mixture of DCM:MeOH, MeOH, Milli-Q water, and 0.1 M NaNO_3_ + 0.01 M H_3_BO_3_ pH 4.0 buffer and stored for approximately 36 h in the same buffer at 4 °C.

## 4. Conclusions

For some time now, electroactive self-assembled monolayers based on various receptors and metals, including dipyrromethenes, have been at the center of our interest [29]. According to our experience, the mixed monolayer of MBL and AHT is well organized and it is efficient for immobilization of terpyridine and complexation with Co(II), followed by closing the coordination sphere by dipyrromethene. Both terpyridines and dipyrromethenes are suitable molecules for creating complexes with transition metal ions. It was reported that terpyridines are π-acceptors and stabilize the metals in a lower oxidation state. These parameters may possibly affect the stability of the entire layer. Our results clearly show that the complex remains stable after an appropriate time of conditioning (7 days of storing). 

The presented electrochemical anion sensing platform contains a chloride-binding receptor as an anion recognition element and a redox-active TPY/Co(II)/DPM complex immobilized on a gold electrode surface. The electrochemical characterization of this sensing layer was assessed using the following electrochemical parameters: electron transfer rate constant (*k*_0_), electron transfer coefficient (*α*) and surface coverage (*Γ*).

The sensor under study enabled sensitive and selective detection of chloride in the fM range in an aqueous medium with a LOD of 0.50 fM. The selectivity of the sensor was evidenced not only by the significantly lower voltammetric response of the sensor to sulfate and bromide but also by an additional parameter: namely, the response ratio, *R_ij_*.

Furthermore, the lack of the need to use an external redox marker makes the sensor more attractive in terms of application and miniaturization. These features make the developed system an interesting analytical tool for monitoring chloride content in the environment.

## Figures and Tables

**Table 1:** Comparison of electrochemical sensors for detection of chloride anions in aquatic media (last decade).

Sensor	Method	LOD, M	Interferents	Ref.
GPE/AgNPs	CV	4.1 × 10^−5^	CO_3_^2−^, NO_3_^−^, NO_2_^−^, PO_4_^3−^, SO_4_^2−^	[36]
CD/Ag	LSV	2 × 10^−5^	NO_2_^−^, PO_4_^2−^, Na^+^, K^+^, SO_4_^2−^	[18]
PTFE/MWCNT/(Ag-NPs/AgCl)/Cu-BTC	ChA	nd	NO_3_^−^, SO_4_^2−^, S^2−^, OH^−^	[19]
GCE/ZIF-8/AgNPs	DPV	6.1 × 10^−7^	NO_3_^−^, SO_4_^2−^, CO_3_^2−^, PO_4_^2−^	[20]
ISE/TCNQ-TTF/CB	Pm	2.51 × 10^−6^	NO_3_^−^, NO_2_^−^, SO_4_^2−^, CO_3_^2−^, HCO_3_^−^, CH_3_COO^−^	[37]
FP/Ag Ink	CV	1 × 10^−3^	HPO_4_^2−^, H_2_PO_4_^2−^, CO_3_^2−^ HCO_3_^−^, Br^−^, I^−^, CN^−^	[38]
OP/Ag Ink	CV	7 × 10^−6^	nd	[39]
ISE/PDMS/AgNWs	Vm	5 × 10^−4^	HCO_3_^−^, CO_3_^2−^, OH^−^, NO_3_^−^	[40]
CSPE/FcMeOH CSPE/K_4_[Fe(CN)_6_] CSPE/K_3_[Fe(CN)_6_]	DPV	0.01 2 2	AsO_4_^2−^, PO_4_^3−^, I^−^, CN^−^, Br^−^, CO_3_^2−^, CrO_4_^2−^, Cr_2_O_7_^2−^, IO_3_^−^, BrO_3_^−^, SO_4_^2−^, Hg_2_^2+^, Pb^2+^	[21]
CSPE/Gore-Tex/FcMeOH	DPV	2 × 10^−4^	nd	[22]
Au/DPM/Cu(II)/DPM-AR Au/DPM/Co(II)/DPM-AR	SWV	1.0 × 10^−12^ 1.1 × 10^−12^	SO_4_^2−^, Br^−^	[23]
Au/AHT,MBL/TPY/Co(II)/DPM-AR	SWV	5.0 × 10^−16^	SO_4_^2−^, Br^−^	This work

Abbreviations: GPE—graphite pencil electrode, AgNPs—silver nanoparticles, GPE—graphite pencil electrode, CD—compact discs, LSV– linear scan voltammetry, PTFE—flexible polytetrafluoroethylene, MWCNT—multiwalled carbon nanotubes, Cu-BTC—hydrophilic copper benzene-1,3,5-tricarboxylate, ChA—chronoamperometry, nd—not determined, ZIF—zeolitic imidazolate framework, DPV—differential pulse voltammetry, ISE—ion-selective electrode, TCNQ-TTF—tetrathiafulvalene-tetracyanoquinodimethane, CB—carbon black, Pm—potentiometry, FP—filter paper, CV—cyclic voltammetry, OP—office paper, PDMS—composite of polydimethylsiloxane, AgNWs—silver nanowires, Vm—voltammetry, CSPE—carbon screen-printed electrode, FcMeOH—ferrocenomethanol, Gore-tex—laminate waterproof breathable ripstop nylon fabric, DPM–AR dipyrromethene- anion receptor, AHT—aminohexanethiol, MBL—mercaptobutanol, TPY—terpyridine, SWV—square wave voltammetry.

## Data Availability

Data will be made available on request.

## Supplementary Materials

The following supporting information can be downloaded at: https://www.mdpi.com/article/10.3390/molecules29092102/s1, Table S1: Response ratio *R_i,j_* of Au/MBL+AHT/TPY-Co(II)/DPM-AR, Figure S1: Redox potential differences Δ*E* = *E_n_*− *E*_0_, mV recorded using SWV for Au/MBL,AHT/TPY/Co(II)/DPM-AR in the presence of chloride. Table S2: The association constant *K_A_*, dissociation constant *K_D_* and Gibbs free energy Δ*G* calculated for gold electrode modified with TPY/Co(II)/DPM-AR. Figure S2: The linear relationship of *I_n_* − *I*_0_*/I*_0_ vs. C_Cl_^−^ [M] for TPY/Co(II)/DPM-AR modified gold electrode surface. Table S3: Binding constant *(K)* and Reaction Coupling Efficiency (*RCE*) of TPY/Co(II)/DPM-AR towards chloride, sulfate and bromide (*n* = 5). Figure S3: Relationship of exp{ΔE°’(-nF/RT)} − 1 vs. concentration of (●) chloride, (■) sulfate and (▲) bromide. Description of anion receptor synthesis. Scheme S1: Synthesis of the dipyrromethene modified dipodal anion receptor.
