# Supplementary material for: Voltammetric Sensing of Chloride Based on a Redox-Active Complex: A Terpyridine-Co(II)-Dipyrromethene Functionalized Anion Receptor Deposited on a Gold Electrode
Source: Molecules. 2024 May 2;29(9):2102. doi: 10.3390/molecules29092102 (PMC11085611; doi:10.3390/molecules29092102)
Supplement: Supplementary file 1 [file molecules-29-02102-s001.zip › molecules-2929252-supplementary.pdf]

Supplementary Materials

Voltammetric sensing of chloride based on a redox active complex: a terpyridine-Co(II)-dipyrromethene functionalized anion receptor deposited on a gold electrode

Kamila Malecka-Baturo[1], Mathias Daniels[2], Wim Dehaen[2], Hanna Radecka[1], Jerzy Radecki [1], Iwona Grabowska [1,]*

[1] *Institute of Animal Reproduction and Food Research, Polish Academy of Sciences, Tuwima 10, 10-748 Olsztyn, Poland; k.malecka@pan.olsztyn.pl (K.M.-B); h.radecka@pan.olsztyn.pl (H.R.); i.grabowska@pan.olsztyn.pl (I.G.)*

[2] *Sustainable Chemistry for Metals and Molecules, Department of Chemistry, KU Leuven, Leuven Chem&Tech, Celestijnenlaan 200F, B-3001 Leuven, Belgium; mathiasdaniels@gmail.com (M.D.); wim.dehaen@kuleuven.be (W.D.)*

* *Correspondence: i.grabowska@pan.olsztyn.pl*

1. The electroactive surface area of gold working electrode A_{eas} was determined by oxygen adsorption measurement and was calculated by equations:

$$\mathrm{A_{eas}} = \frac{\mathrm{Q_{Au}}}{\mathrm{Q}_H^S} = 0.32 \pm 0.02\ \mathrm{cm}^2 \quad \text{(S1)}$$

$$Q_{Au} = \frac{A_{p,c}}{v} \quad \text{(S2)}$$

where $A_{p,c}$ is the charge of the gold oxide reduction of the gold working electrode, v is scan rate (0.1 V/s), and Q_H^S is the standard`s reference charge (0.00039 ± 0.00001 C/cm^2) suggested for polycrystalline gold [1].

2. The roughness factor (*RF)* was calculated according to equation:

$$RF = \frac{A_{\mathrm{aes}}}{A\mathrm{geom}} = 10.34 \pm 0.52, \text{ where } \mathrm{A_{geom}} = 0.0314\ \mathrm{cm}^2 \quad \text{(S3) [2]}$$

3. Calculation procedure for surface coverage Γ [mol/cm^3]

Based on the slopes of the relationships presented in Fig. 1B, the surface coverage (Γ) was calculated using the following equation:

$$I_p = \frac{n^2 F^2 v A \Gamma}{4RT} \quad \text{(S4) [3]}$$

where n – the number of electrons involved in the oxidation or reduction process; $F = 96500\ C\ mol^{-1}$ – the Faraday's constant; ν – scan rate; A – is the area of the electrode; R – ideal gas constant; T – the temperature in Kelvin.

4. Calculation procedure for α and k $[s^{-1}]$

To determine the electron transfer coefficient α, the peak potential E_p is plotted *vs.* log ν [4]. E_{pa} and E_{pc} are plotted separately in this way to give two branches. The slope of the line is given in equation:

$$slope = -\frac{2.3RT}{\alpha nF} \quad \text{(S5)}$$

Determining the x-intercepts of the lines for anodic and cathodic branches provides ν_a and ν_c, respectively, values that are used in Eq. S5 to determine the electron transfer rate constant k_0 [5]:

$$k = \frac{\alpha nF\vartheta_c}{RT} = \frac{(1-\alpha)nF\vartheta_a}{RT} \quad \text{(S6)}$$

where: n is the number of electrons involved in the oxidation or reduction process; $F = 96500$ [C mol^{-1}] – the Faraday constant; $R = 8.3145$ [$J\ mol^{-1}K^{-1}$] is the ideal gas constant; $T = 273K$ – room temperature.

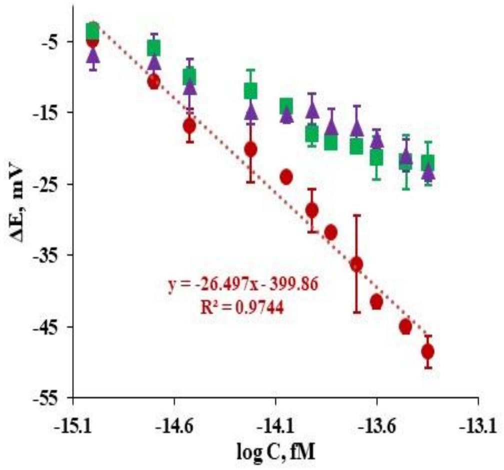

Figure S1: Redox potential differences $\Delta E = E_n - E_0$, mV recorded using SWV for Au/MBL,AHT/TPY/Co(II)/DPM-AR in the presence of chloride.

5. Selectivity $R_{i,j}$ was calculated based on the slope ratio as recommended by Macca and Wang [6], equation:

$$R_{i,j} = \frac{S_j}{S_i} \quad \text{(S7)}$$

where: S_i is the slope of the calibration curve for chloride; S_j is the slope of the calibration curve for sulphate/bromide.

6. The selectivity factor α_f of the proposed sensor was estimated by the Equation:

$$\alpha_f = \frac{\left(\frac{\Delta I_n}{I_0}\right)_{Cl-}}{\left(\frac{\Delta I_n}{I_0}\right)_{Int}} \quad \text{(S8)}$$

where $\left(\frac{\Delta I_n}{I_0}\right)_{Cl-}$ and $\left(\frac{\Delta I_n}{I_0}\right)_{Int}$ are normalized responses of the Au/ MBL,AHT/TPY/Co(II)/DPM-AR to Cl^- and interfering agents (sulphate and bromide) [7].

7. Limit of detection (*LOD*) was calculated using *IUPAC* (International Union of Pure and Applied Chemistry) definitions according to the equation:

$$LOD = \frac{3.3\sigma}{q} \quad \text{(S9)[8]}$$

where σ is the standard deviation of the response and q is the slope of the calibration curve.

Table S1: Response ratio $R_{i,j}$ of the Au/MBL+AHT/TPY-Co(II)/DPM-AR sensor.

Anion	$R_{i,j}$
Cl^-	1
SO_4^{2-}	0.73
Br^-	0.47

8. The association constant (K_A) of DPM-AR with chloride was calculated using a Langmuir isotherm approach [9].

$$k_A C = \frac{I_n - I_0}{I_0} \quad \text{(S10)}$$

where, C is a concentration of molecules in the solution, and I_0 and I_n mean the peak currents without and in the presence of a particular concentration of chloride, respectively.

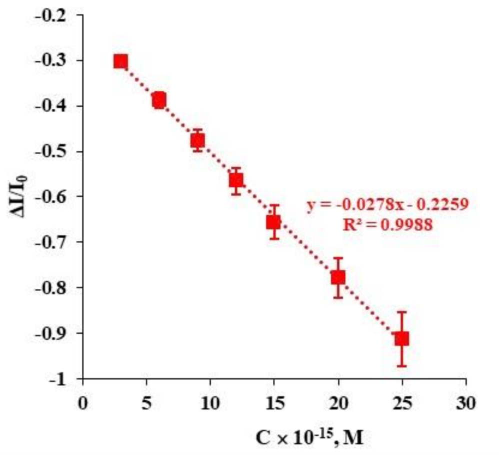

Figure S2: The linear relationship of $(I_n - I_0/I_0)$ *vs.* C_{Cl^-} [M] for TPY/Co(II)/DPM-AR modified gold electrode surface.

Based on the obtained results, the dissociation constant K_D was calculated according to the following relationship:

$$K_A = \frac{1}{K_D} \quad \text{(S11)}$$

9. Gibbs free energy (ΔG) was calculated using the Van't Hoff equation:

$$\Delta G = -RT ln K_A \quad \text{(S12)[10]}$$

Table S2: The association constant K_A, dissociation constant K_D and Gibbs free energy ΔG calculated for a gold electrode modified with TPY/Co(II)/DPM-AR.

K_A [M^{-1}]	K_D [M]	ΔG [kJ/mol]
$2.78 \pm 0.21 \times 10^{13}$	$6.13 \pm 0.45 \times 10^{-14}$	-69.6 ± 2.0

10. The interface binding constants (K) were estimated according to the procedure reported by Gobi and Ohsaka [11] based on equation:

$$exp\{\Delta E°'(-nF/RT)\} - 1 = K_1[X] + K_1K_2[X]_2 \quad \text{(S13)}$$

where R, F, and T (K) have their usual meaning, n is the number of electrons transferred (in the present case, it is equal to one), and [X] represents the concentration of target anions.

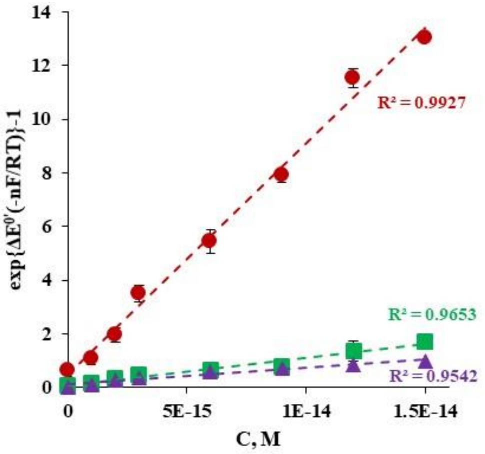

Figure S3: Relationship of *exp{ΔE°'(-nF/RT)}–1 vs.* concentration of (●) chloride, (■) sulfate and (▲) bromide.

The formal potential ($E°'$) values were estimated as the average of anodic and cathodic peak potentials. The averages of the anodic and cathodic potential shifts ($\Delta E°'$) were calculated from the CV curves for each anion concentration, and the respective $exp\{\Delta E°'(-nF/RT)\}-1$ values were plotted *vs.* the concentrations of the anions. The corresponding graphs in Fig. S3 show that the relationship is linear, and the slopes accordingly provide access to the interface binding constants of the system under study.

11. In order to confirm the good selectivity of the sensor towards chloride, the reaction coupling efficiency (*RCE*) was calculated using an equation [12]:

$$RCE = \frac{K_1}{K_2} = e^{\frac{nF\Delta E^0}{RT}} \qquad (S14)$$

where, K_1 and K_2 are the stability constants of the complex in different redox states, and ΔE^0 is the magnitude of the electrochemical shift.

Table S3: Binding constant (*K*) and Reaction Coupling Efficiency (*RCE*) of the MBL,AHT/TPY/Co(II)/DPM-AR towards chloride, sulfate and bromide (n = 5).

Parameters	Cl^-	SO_4^{2-}	Br^-
$K{\times}10^{+13}[M^{-1}]$	20.00	5.13	2.13
RCE	5.2 ± 0.5	2.3 ± 0.6	1.3 ± 0.3

Description of anion receptor synthesis

The synthesis scheme of anionic receptor modified with dipyrromethene ((Z)-4-((1H-Pyrrol-2-yl)(2H-pyrrol-2-ylidene)methyl)-N-(2-(bis(2-(3-hexylureido)ethyl) amino)ethyl)benzamide) is presented on **Scheme S1**. In brief, the compound **1** (Tris-2-aminoethylamine) was mono Boc-protected in dichloromethane according to a previously reported procedure [13] and the compound **2** was formed. The reaction between compound **2** with n-hexyl isocyanate resulted in formation of compound **3**, followed by removing of the Boc protecting group with trifluoroacetic acid in order to obtain compound **4.** The preparation of compound **8** was done by hydrolysis of dipyrromethene methyl ester **7** according to the procedure reported previously using compounds number **5** and **6** [14]. The compound **9 –** activated NHS ester was obtained via a DDC mediated coupling reaction in the presence of DMAP. The final step concerned the reaction between compound **9** and **4** and the dipodal anion receptor **10** was formed. The detailed synthesis of dipodal anion receptor (compound **10**) is described in the manuscript by Kaur and co-workers [15].

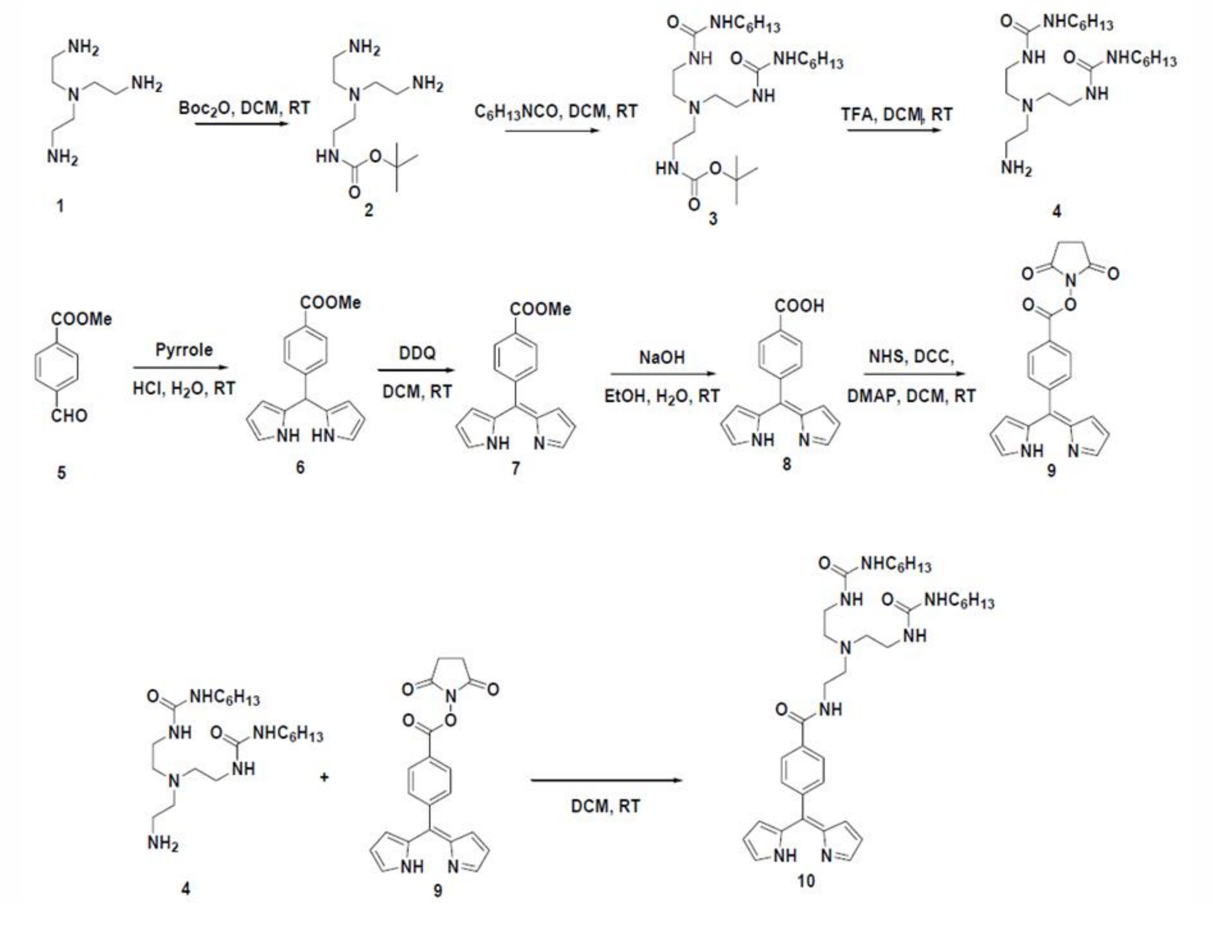

Scheme S1. Synthesis of the dipyrromethene modified dipodal anion receptor.

References

1. Zaki, M. H. M.; Mohd, Y.; Chin, L. Y., Surface Properties of Nanostructured Gold Coatings Electrodeposited at Different Potentials. *International Journal of Electrochemical Science* **2020,** 15, (11), 11401-11415.
2. Xie, X.; Holze, R., Electrode Kinetic Data: Geometric vs. Real Surface Area. *Batteries* **2022,** 8, (10), 146.
3. Bard, A. J.; Faulkner, L. R.; White, H. S., *Electrochemical methods: fundamentals and applications*. John Wiley & Sons: 2022.
4. Laviron, E., General expression of the linear potential sweep voltammogram in the case of diffusionless electrochemical systems. *Journal of Electroanalytical Chemistry and Interfacial Electrochemistry* **1979,** 101, (1), 19-28.
5. Eckermann, A. L.; Feld, D. J.; Shaw, J. A.; Meade, T. J., Electrochemistry of redox-active self-assembled monolayers. *Coord Chem Rev* **2010,** 254, (15-16), 1769-1802.
6. Maccà, C.; Wang, J., Experimental procedures for the determination of amperometric selectivity coefficients. *Analytica Chimica Acta* **1995,** 303, (2), 265-274.
7. Amouzadeh Tabrizi, M.; Acedo, P., An Electrochemical Immunosensor for the Determination of Procalcitonin Using the Gold-Graphene Interdigitated Electrode. *Biosensors* **2022,** 12, (10), 771.
8. Swartz, M. E., & Krull, I.S. (Eds.). .. , Analytical Method Development and Validation (1st ed.). CRC Press: (1997).
9. Szymańska, I.; Stobiecka, M.; Orlewska, C.; Rohand, T.; Janssen, D.; Dehaen, W.; Radecka, H., Electroactive Dipyrromethene−Cu(II) Self-Assembled Monolayers: Complexation Reaction on the Surface of Gold Electrodes. *Langmuir* **2008,** 24, (19), 11239-11245.
10. Sikarwar, B.; Singh, V. V.; Sharma, P. K.; Kumar, A.; Thavaselvam, D.; Boopathi, M.; Singh, B.; Jaiswal, Y. K., DNA-probe-target interaction based detection of Brucella melitensis by using surface plasmon resonance. *Biosens Bioelectron* **2017,** 87, 964-969.
11. Gobi, K. V.; Ohsaka, T., Anion recognition and electrochemical characteristics of the self-assembled monolayer of nickel(II) azamacrocyclic complex. *Journal of Electroanalytical Chemistry* **2000,** 485, (1), 61-70.
12. Beer, P. D.; Gale, P. A.; Chen, Z., Electrochemical Recognition of Charged and Neutral Guest Species by Redox-active Receptor Molecules††Manuscript received 9th October 1995. In *Advances in Physical Organic Chemistry*, Bethell, D., Ed. Academic Press: 1999; Vol. 31, pp 1-90.
13. Rohand, T.; Dolušić, E.; Ngo, H. T.; Maes, W.; Dehaen, W., Efficient synthesis of aryldipyrromethanes in water and their application in the synthesis of corroles and dipyrromethenes. *Alain Krief ARKIVOC* **2007,** 2007, 307-324.
14. Boon, J. M.; Lambert, T. N.; Smith, B. D.; Beatty, A. M.; Ugrinova, V.; Brown, S. N., Structure/Activity Study of Tris(2-aminoethyl)amine-Derived Translocases for Phosphatidylcholine. *The Journal of Organic Chemistry* **2002,** 67, (7), 2168-2174.
15. Kaur, B.; Erdmann, C. A.; Daniëls, M.; Dehaen, W.; Rafiński, Z.; Radecka, H.; Radecki, J., Highly Sensitive Electrochemical Sensor for the Detection of Anions in Water Based on a Redox-Active Monolayer Incorporating an Anion Receptor. *Analytical Chemistry* **2017,** 89, (23), 12756-12763.
