# Supplementary figures and images for: Voltammetric Sensing of Chloride Based on a Redox-Active Complex: A Terpyridine-Co(II)-Dipyrromethene Functionalized Anion Receptor Deposited on a Gold Electrode
Source: Molecules. 2024 May 2;29(9):2102. doi: 10.3390/molecules29092102 (PMC11085611; doi:10.3390/molecules29092102)

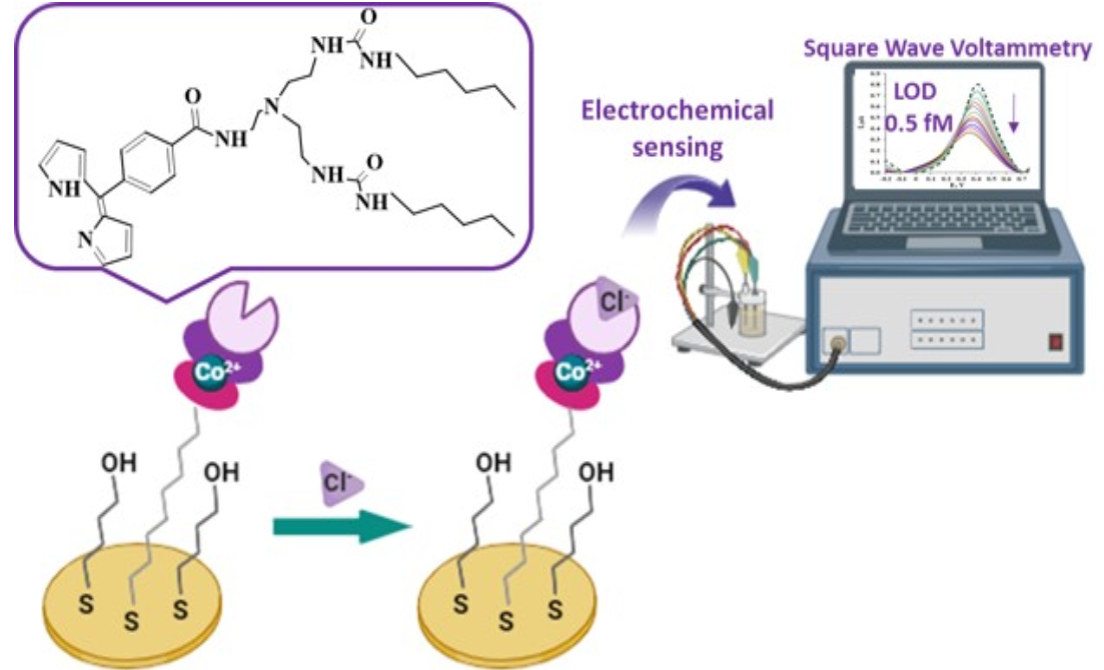

Supplement: Supplementary file 1 [file molecules-29-02102-s001.zip › molecules-2929252-graphical.png]
